# Updates in interventional cardiology in children with cardiac disease

**DOI:** 10.1007/s00431-025-06236-z

**Published:** 2025-06-07

**Authors:** Kamel Shibbani, Damien Kenny

**Affiliations:** 1 University of Iowa Children’s Hospital, Iowa, USA; 2 Our Lady’s Children’s Hospital, Crumlin, Dublin Ireland

**Keywords:** Cardiac disease, Congenital heart disease, Transcatheter interventions

## Abstract

The field of pediatric and congenital interventional cardiology is advancing rapidly, with recent innovations such as patent ductus arteriosus closure in premature infants, expanded options for transcatheter pulmonary valve replacement, ductal stenting for ductal-dependent lesions, and the development of novel, pediatric-specific devices. Despite these advancements, significant variability in care persists across centers, largely due to the absence of standardized guidelines. This gap can be addressed through the use of registries and multicenter studies, which offer evidence-based insights into optimal management strategies for congenital heart disease.

*Conclusion*: This review aims to spotlight emerging procedures and devices used in the catheterization lab, presenting clinically relevant data on cutting-edge transcatheter interventions. Additionally, we discuss areas of ongoing debate and propose future research directions to further refine and standardize pediatric interventional cardiology practices.

**Table:** 

**What is Known:** *• Transcatheter procedures in congenital heart disease are growing in breadth and complexity.*	
**What is New:** *• There is significant variability in management strategies within pediatric interventional cardiology that can be addressed using registries and multicenter studies.* *• Novel devices like new self-expanding pulmonary valves, bioresorbable stents, and pediatric specific devices are being developed.*

**What is Known:**

*• Transcatheter procedures in congenital heart disease are growing in breadth and complexity.*

**What is New:**

*• There is significant variability in management strategies within pediatric interventional cardiology that can be addressed using registries and multicenter studies.*

*• Novel devices like new self-expanding pulmonary valves, bioresorbable stents, and pediatric specific devices are being developed.*

## Introduction

The first description of the cardiovascular system as one that consists of two parallel but separate circulations was published in 1628 by William Harvey. More than 300 years later, in 1938, Robert Gross performed the first pediatric cardiac surgery by ligating a ductus arteriosus in a 7-year-old child [1]. Only 30 years later, in 1966, Rashkind and Miller first reported the creation of an atrial septal defect without a thoracotomy in an unanesthetized infant [2]. In a 1998 review article written by Charles Mullins, widely considered to be the father of modern pediatric interventional cardiology, he mused that “The most recent effective new development in pediatric and congenital therapeutic catheterizations … involves the opening of vessels” through stent angioplasty of the pulmonary arteries. It would only take 2 years and 4 months from the date of publication of that review article [3] for the publication of the first transcatheter pulmonary valve replacement in a pediatric patient [4]. The pace of advancement in interventional cardiology has been exponential. Herein, we describe the most recent advances in the field of pediatric and congenital interventional cardiology, covering patent ductus arteriosus stenting, transcatheter pulmonary valves, novel pediatric-specific designed devices, and more.

## Ductal and right ventricular outflow tract (RVOT) stenting

As early as 1991, ductal stenting had been recognized as a potential desirable alternative to surgical shunts [5] [6], especially in the setting of ductal-dependent pulmonary circulation. Initial widespread use of the procedure was limited by the lack of appropriate delivery and stent platforms [7], the potential for catastrophic complications like ductal spasm and stent thrombosis [8], as well as the technical difficulty of performing the procedure in patients who are often ductal dependent. However, the last decade has seen a growing acceptance of ductal stenting in lieu of a surgical shunt. The largest retrospective study comparing ductal stenting to surgical shunts in ductal-dependent pulmonary circulation was performed by Glatz et al. [9]. This was a multicenter study that included 357 patients from 4 North American sites. It showed that ductal stenting is associated with improved pulmonary artery size and symmetry, decreased ICU length of stay, and a trend towards decreased hospital length of stay. There were no differences in mortality or rates of unplanned reintervention [9]. A simultaneous retrospective European study by Bentham et al. [10] which included 254 patients from 9 centers in the UK also showed encouraging findings with decreased risk of death before repair and increased overall survival. Unlike the Glatz et al. study, however, the Bentham et al. data showed a higher rate of reintervention. Similarly, several meta-analysis studies comparing ductal stenting to surgical shunts have by and large shown that ductal stenting is noninferior in clinical outcomes to surgical shunts [8, 11].

One of the areas that is garnering more attention in this space is the effect of ductal anatomy on the success of the procedure. Determining the size and length of the stent can be challenging, especially in ductal-dependent pulmonary circulation where the duct tends to be tortuous. Despite the utility of pre-procedural computed tomography (CT) scans in planning for ductal stenting [12–14], the tortuosity of the duct continues to present challenges in predicting the appropriate stent length. A stent that is too short can lead to significantly diminished pulmonary flow if the unstented portion of the duct constricts, while a stent that is too long can present a significant challenge for the surgeon during repair.

Right ventricular outflow tract stenting has also been shown to be a safe and effective strategy for increasing pulmonary blood flow, albeit using data from retrospective single-center studies. This strategy has predominantly been used to delay primary Tetralogy of Fallot (TOF) repair in infants. Forty-two patients from Canada [15] and 20 patients from Australia [16] with TOF underwent RVOT stenting, with successful delay in repair to a median of 6 months in both studies.

## Patent ductus arteriosus (PDA) closure in premature infants

**Fig. 1:**
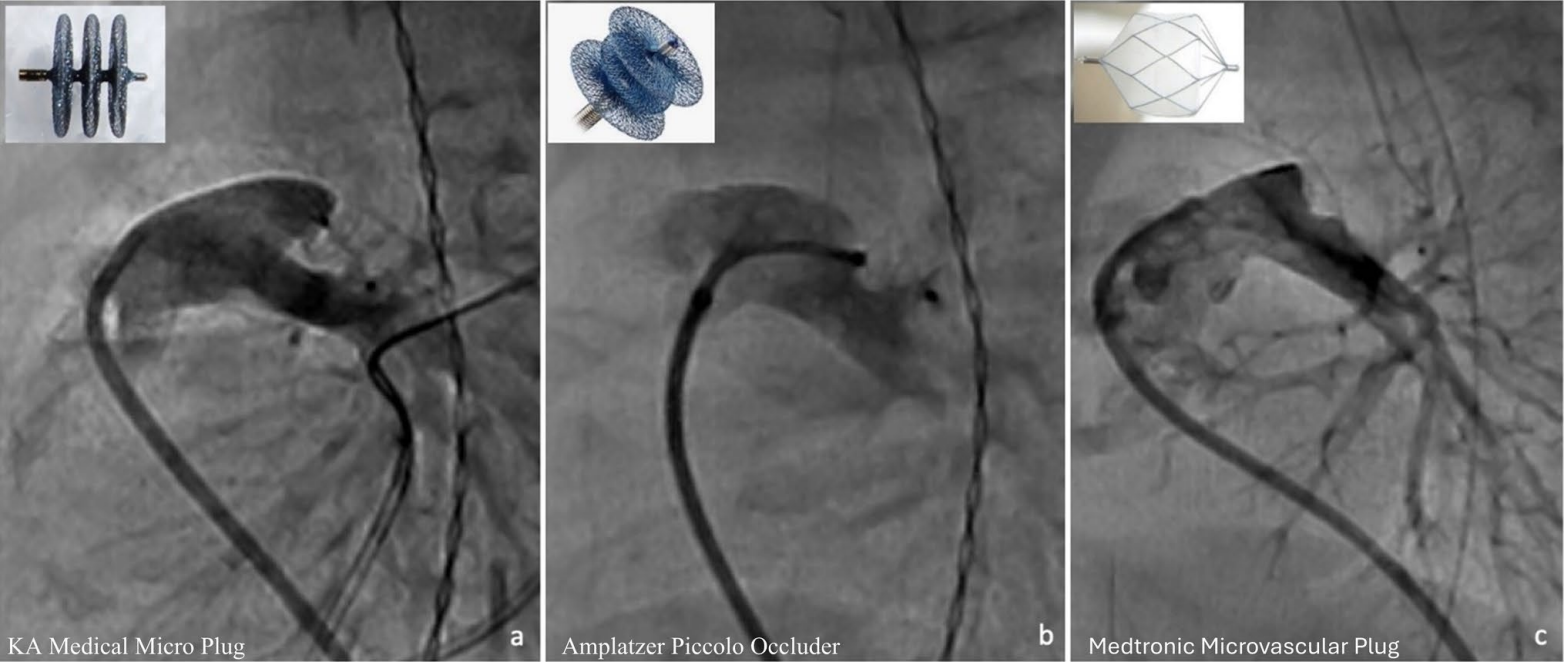
Devices used for PDA closure in extremely low birth weight premature infants. **a** The KA Micro plug has three equal sized disks. **b** the Piccolo is the only device with FDA approval and CE Marking for PDA closure in premature infants and is made from 2 equal sized disks on either end of the device with a central disk of variable length in the middle. **c** The microvascular plug has an ovoid shape. This is the same device that is also used in pulmonary flow restrictors

In premature infants, the PDA has been associated with increased morbidity and mortality. Various studies have shown an association between a PDA and intraventricular hemorrhage, necrotizing enterocolitis, bronchopulmonary dysplasia, and death [17–20]. Given these findings, PDA closure in premature infants has been an area of much interest, with randomized trials for closure, albeit surgical, dating back to 1989 [21]. Currently, the Amplatzer Piccolo Occluder (Abbott Structural Heart, CA) is the only device that has FDA approval and CE marking. This device is approved for infants ≥ 3 days of age and weighing ≥ 700 g. Several other devices have been commonly used off-label, the most frequent being the Medtronic microvascular plug ((MVP); Medtronic, MN) and the KA Medical Micro Plug (KA Medical, MN) (Fig. 1). The largest retrospective multicenter comparative study that looked at all three devices included 287 patients. It found them all to be equally efficacious and safe, with no reported difference in mortality or morbidity associated with the procedure, and with an overall successful rate of PDA closure of 98% [22].

Despite the trend towards more aggressive PDA closure [23], there continues to be a debate about the utility of addressing the PDA in premature infants. Indeed, two randomized trials out of Europe have shown no significant benefit to early pharmacological therapy. The BeNeDuctus trial was a randomized non-inferiority trial that included 273 patients and compared the rates of death, necrotizing enterocolitis (NEC), and bronchopulmonary dysplasia (BPD) in patients undergoing expectant management to those receiving medical management for PDA closure [24]. The trial found expectant management to be non-inferior. Similarly, the baby-OSCAR trial was a multicenter, masked, randomized placebo-controlled trial that found no difference in the risk of death or BPD between the early treatment and placebo groups [25].

## Transcatheter pulmonary valve replacement (TPVR)

One of the areas of congenital interventional cardiology that has received the most attention in recent years has been TPVR. Traditionally, TPVR in congenital heart disease patients had been restricted to the use of balloon expandable valves, namely the Melody (Medtronic) and the Sapien (Edwards) valves. Balloon expandable valves oftentimes cannot accommodate a dilated native right ventricular outflow tract that is present in up to 80% of patients with congenital heart disease in need of TPVR [26]. Recently, self-expanding platforms designed to accommodate dilated native right ventricular outflow tracts have become available, making it feasible to deploy the valve in a larger diameter vessel.

The Melody valve is composed of a bovine jugular vein that is sutured onto a platinum-iridium stent frame. The Melody valve is available in three sizes, 18 mm, 20 mm, and 22 mm [26]. The Sapien valve, on the other hand, is composed of bovine pericardium that is shaped into three leaflets and mounted on a cobalt-chromium stent [26]. There are multiple iterations of the Sapien valve, with the Sapien 3 being the most commonly used iteration for TPVR. The Sapien 3 is available in 19, 23, 26, and 29 mm sizes [27, 28].

**Fig. 2:**
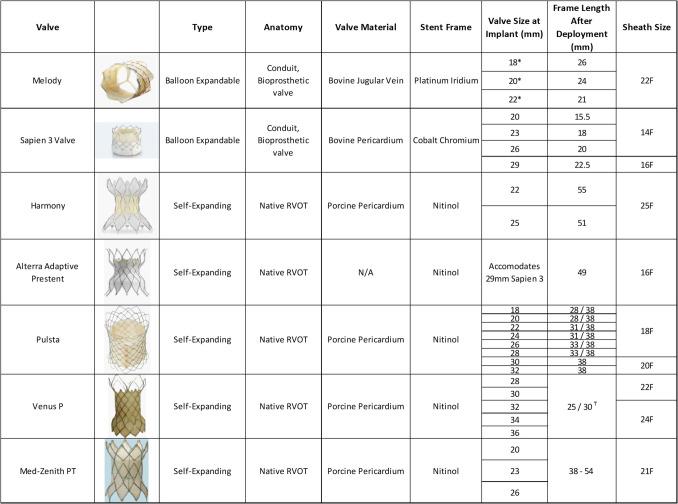
Common transcatheter pulmonary valve replacement options. Adapted with permission from Patel ND, Levi DS, Cheatham JP, Qureshi SA, Shahanavaz S, Zahn EM. Transcatheter pulmonary valve replacement: a review of current valve technologies. J Soc Cardiovasc Angiogr Interv. 2022;1(6):100452. Published 2022 Sep 16. https://doi.org/10.1016/j.jscai.2022.100452. *The diameter of the Melody valve refers to the inner diameter; the outer diameter is ~ 2 mm larger for each respective valve. ₸ 25/30 mm refers to the length of the central unflared section

Self-expanding platforms allow TPVR in larger diameter native RVOT. Such platforms include the Harmony (Medtronic), the Alterra Prestent (Edwards), the Pulsta (TaeWoong Medical Co), the Venus P (Venus Medtech), and the Med-Zenith PT valve (Beijing Med-Zenith). The Harmony valve is available in two sizes, the TPV23 and the TPV25, which refer to the valve size that is housed within the stent frame. It has a nitinol frame and the valve itself is composed of porcine pericardium. The Alterra Prestent platform is a nitinol frame covered with PTFE that is deployed in the right ventricular outflow tract (RVOT) and is designed to accommodate a Sapien 3 29-mm valve that is delivered separately [29]. All three remaining self-expanding valves, the Med-Zenith PT, the Pulsta, and the Venus P valve, are made from a nitinol frame covered with porcine pericardium and use porcine pericardium for valve material as well (Fig. 2).

Although different guidelines exist with various thresholds for intervention, TPVR is generally indicated for significant RV dysfunction in the setting of regurgitation or stenosis (Table 1) [30–36]. The ideal timing of TPVR remains unclear and is an area of active research. While most criteria rely on severe dilation of the RV or the presence of symptoms, there is a strong case to be made that TPVR should be started before such metrics are reached. Indeed, some studies have shown that up to 54% of patients with RV dysfunction continue to have some degree of RV impairment after TPVR [37]. The decision of early TPVR needs to be balanced against the obvious consequence of an increase in the number of procedures per patient if early TPVR is universally applied.

**Table 1:** Criteria for TPVR

Valve	Location	Criteria	Study
Sapien	Conduit or surgical valve	Significant regugitation (≥ 3 + by echo, regurgitant fraction ≥ 40% by MRI) OR significant stenosis (RVOT mean gradient of ≥ 35 mmHg)	Compassion Trial [31]
Melody	Conduit or surgical valve	(NYHA class II, III, IV) AND [(mean gradient across the RVOT of 35 mmHg) OR (moderate to severe regurgitation)]	IDE Trial [32]
		(NYHA class I) AND [(Mean gradient ≥ 40) OR (Severe regurgitation)]	
Harmony	Native RVOT	[(Regurgitation fraction ≥ 30% by MRI) OR (Severe PR by echo)] AND [(RVEDVi ≥ 150 ml/m^2^) OR (Symtpoms)]	Harmony Feasibility Trial [33]
Pulsta	Native RVOT	(Moderate or severe regurgitation) AND (RVEDi ≥ 150 ml/m^2^)	Pulsta Feasibility Trial [34]
Venus P valve	Native RVOT	(Severe regurgitation by echo) AND (Regurgitant fraction ≥ 30% by MRI) AND (RVEDVi of ≥ 150 ml/m^2^) AND Symtpoms	Venus P-valve Early Results [35]
Med-Zenith PT	Native RVOT	(NYHA class II, III, IV) AND [(Regurgitation fraction ≥ 30% by MRI) OR (Regurgitation ≥ 3 by echo)]	Med-Zenith PT-Valve clinical Trial Outcomes [36]
		(NYHA class I) AND [(RVEDVi of ≥ 150 ml/m2) OR (RVEDVi:LVEDVi) ≥ 2]	

The long-term outcomes for TPVR are more readily available for the older balloon expandable valves. The Melody valve has excellent long-term survival of 90% with freedom from reintervention of 60%. The Melody valve, however, has a rate of endocarditis that is estimated at 2% per year [32]. The Sapien valve may be less prone to infective endocarditis, and comparison of 10-year data between the Melody and the Sapien showed that freedom from secondary pulmonary replacement was higher in the Sapien group (82.2% vs 50.4%) [38]. Indeed, recent data shows that the 6-year cumulative incidence of infective endocarditis for the Sapien 3 valve was 3.8% [39]. The newer self-expanding valves lack the robust long-term data available for their balloon expandable counterparts, though early data is encouraging for safety and need for reintervention [29, 40, 41]. It is worth noting that self-expanding platforms are associated with a higher incidence of periprocedural ventricular arrhythmia as compared to balloon expandable platforms, with early data suggesting that non-sustained ventricular tachycardia occurs in up to 40% of self-expanding TPVR in the 24 h after deployment [42]. Multivariate analysis showed that a pre-procedural diagnosis of pulmonary stenosis was associated with a higher risk of ventricular tachycardia, with a supra-annular implant being borderline protective [43]. Though anti-arrhythmics have been used to mitigate against ventricular tachycardia post TPVR, there is no data to suggest that these medications decrease the incidence or morbidity of arrhythmia post TPVR.

Finally, Ribeiro et al. [44] performed a meta-analysis that compared outcomes of TPVR to surgical pulmonary valve replacement (SPVR). This study included 6071 patients, with 1132 patients having undergone TPVR and 4939 having undergone SPVR. The study found no difference in mortality or reintervention rate, shorter hospital stays for TPVR, and higher rates of infective endocarditis in TPVR. These findings were largely echoed by Zhou et al. [45] in their meta-analysis that included 4364 patients and found a decreased hospital stay associated with TPVR and increased infective endocarditis associated with TPVR. Interestingly, Zhou et al. [45] reported lower in-hospital mortality and mortality at the latest follow-up in the TPVR group.

## Sinus venosus atrial septal defect (ASD) closure

Although surgical closure of a sinus venosus atrial septal defect (SV-ASD) continues to be the standard of care, transcatheter closure can now be offered in patients that are deemed to be poor surgical candidates. This highly specialized procedure involves the use of covered stents to close a SV-ASD and re-route right upper pulmonary venous (RUPV) drainage into the LA and was first performed in 2011 [46]. The procedure has had several modifications, including the use of a temporary stay suture to secure the stent during deployment, the use of a second anchoring bare metal stent, and the creation of a small atrial-level communication through the septum secundum to cannulate and balloon the RUPV during stent deployment to avoid RUPV compression [47, 48]. Long-term follow-up for patients undergoing percutaneous closure of SV-ASD is encouraging, with small case series showing complete or near complete occlusion for up to 6 years of follow-up [46].

## Atrio-ventricular valve (AVV) interventions

Isolated AVV abnormalities requiring intervention are less common in pediatric and congenital heart disease patients than in adults. However, when AVV abnormalities are present as part of a broader congenital heart disease diagnosis, they can lead to significant morbidity. Despite having a large impact on management strategies and outcomes, percutaneous options for AVV repair or replacement have generally been limited. This paradigm is beginning to shift, with recent experience pointing to the feasibility of both replacement and repair of AVV in patients with congenital heart disease. Maschietto et al. published a case series of eight pediatric patients ranging between 7 and 15 years of age who underwent successful transcatheter mitral valve replacement using a Sapien S3 valve [49]. Although the median follow-up was only 9 months, all procedures were successful with only one document reintervention. Patients with mitral stenosis had significant improvement in gradients across the valve, and no patient had any significant regurgitation [49]. An alternative to replacement is AVV repair, with the adoption of transcatheter edge-to-edge repair (TEER) from adults to pediatric patients. Jolley et al. reported the use of TEER in four patients with at least severe systemic AVV regurgitation [50]. The MitraClip system was successfully deployed in all four patients, though the degree of improvement in regurgitation was variable. Still, this provides proof of concept for the adoption of a technique that is predominantly used in adults. Similarly, Guerin et al. have successfully used the MitraClip system to reduce regurgitation, though they published work done in patients with a systemic tricuspid valve [51]. They reported this technique in 11 patients with systemic right ventricles and showed that there was clinical and echocardiographic improvement in the tricuspid regurgitation, though the procedure was not without its challenges.

## Novel devices

### Renata-Minima

**Fig. 3:**
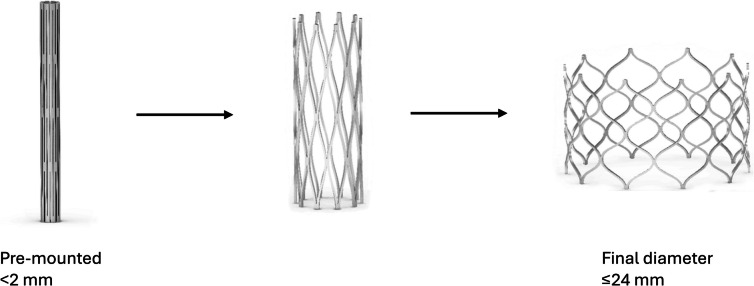
Renata Medical Minima stent. The Minima stent is deployed through a 4 Fr sheath-equivalent delivery system and can be dilated to 24 mm, obviating the need to fracture and re-stent in growing patients

There is a relative paucity of devices specifically designed for pediatric congenital heart disease patients. In an exciting development, the FDA recently approved the Minima Stent System (Renata Medical), with a plan to apply for CE Mark in 2026. The Minima stent is a balloon-mounted cobalt-chromium stent capable of achieving diameters ranging from 4 to 22 mm while maintaining stent integrity and radial strength over the entire range of diameters, albeit with significant foreshortening at larger diameters [52] (Fig. 3). Most impressively, the delivery system used to deploy the stent is equivalent to a 4 Fr sheath in diameter [52].

### Fenestrated atrial septal defect device (f-ASD)

Another device to make its way into the congenital interventional field is the f-ASD device by Occlutech. The device is available with fenestrations ranging in diameter from 4 to 10 mm in 2 mm increments, and with distances of 2, 5, and 10 mm between the left and right atrial disks. The presence of a stable intra-atrial communication is of benefit to patients with significant ventricular diastolic dysfunction, congestive heart failure [53, 54], significant pulmonary hypertension [55–57], and those with severely elevated atrial pressures. In such instances, the ability to introduce a manufactured fenestration allows for precise amounts of flow across the atrial septum, which can be tailored to the desired saturation or flow effect [58]. The device has been shown to be safe and efficacious with documented short-term hemodynamic and symptomatic improvement in patients with various forms of congenital heart disease [59].

### Bioresorbable stents

The potential benefits of bioresorbable stents in pediatric patients with congenital heart disease have led to significant research into this technology. Materials used include iron, zinc, magnesium, and polymers such as poly-l-lactic acid. Zinc-based stents have relatively inert byproducts but provide weak radial strength, while magnesium- and iron-based stents generally have better radial strength but produce byproducts that persist in the vessels and may even limit future MRI use [60]. Not infrequently, this technology has faltered, with the FDA removing the Bioresorbable Vascular Scaffold from the market in 2017 due to device thrombosis [61]. Pediatric-specific stents and stents geared for the congenital field are slowly making their way to the market. Examples include the IBS Angel, the Magmaris, the ZeBRa, and the Freesolve. The use of these stents remains limited in part due to the lack of approval from regulatory bodies and in part due to the mixed results noted with published data. For example, the Magmaris stent has been undermined by rapid degradation in several studies [62, 63] and significant in-stent stenosis has been reported with the IBS Angel stent [64].

### Flow restrictors

Transcatheter pulmonary flow restrictors allow for a percutaneous stage 1 palliation through simultaneous PDA stenting and transcatheter limitation of pulmonary blood flow. Multiple devices have been used, including a fenestrated Amplatzer ASD occluder, a diabolo-shaped covered stent, and, most commonly, a fenestrated micro vascular plug device (Medtronic, Minneapolis, MN). The use of micro vascular plug devices as pulmonary flow restrictors has been found to be safe and effective [65–67], though cases of distal device migration, pulmonary over-circulation, pulmonary artery stenosis, and jailing of the branch pulmonary artery have been documented [68, 69].

## Radiation/imaging advances

Children are at an increased risk of developing adverse outcomes from radiation exposure due to several factors including the need for repeated catheterizations, the increased complexity of congenital heart disease that often requires long procedures, and the close proximity of chest organs to the heart in small children [70]. This results in a well-documented increase in lifetime attributable risk of cancer in pediatric patients undergoing cardiac catheterization [71], as well as to the catheterization lab staff involved in their care [72]. Consequently, there is a continuous need to minimize radiation exposure in the catheterization lab. The utilization of newer imaging platforms that offer improved image resolution at a fraction of the previous ionizing radiation dose is one such way to decrease radiation exposure [73]. In addition, 3-D rotational angiography (3DRA) has been proposed as an option to minimize radiation exposure from CT scans. 3DRA leads to the acquisition of rich data sets that can then be used during the case to guide complex procedures, and they can be obtained at a fraction of the radiation dose that a CT scan delivers [74]. One other possibility is the use of magnetic resonance imaging in real-time to perform MRI-guided catheterization (iCMR). The benefits of iCMR include the complete elimination of ionization radiation, the elimination of iodine-based contrast, and the ability to obtain a more robust hemodynamic dataset by combining both MRI and catheterization-based pressure, flow, and volume assessment [75]. iCMR, however, is not without its challenges. The use of MRI compatible wires, catheters, and devices that are not ferromagnetic is one such challenge that currently restricts the applications of iCMR [75]. The creation of an interventional cardiac MRI suite is also cost- and space-prohibitive for many centers and requires the recruitment of MRI technologists as additional personnel. Lastly, there is a steep learning curve with iCMR cases and even simple diagnostic cases can take several hours at first.

## Data registries

A consensus statement has been recently set forth that addresses the organization of pediatric and congenital catheterization laboratories, procedural and training competencies, facility requirements, and many aspects of the perioperative management of pediatric and adult patients with congenital heart disease [76]. The authors highlight the importance of external comparison and national dataset reporting, such as the use of data registries to track outcomes, identify risk, and improve processes. Examples include the Congenital Cardiovascular Interventional Study Consortium (CCISC), the Congenital Cardiac Catheterization Project on Outcomes (C3PO), the Improving Pediatric and Adult Congenital Treatment (IMPACT), the National Institute of Cardiovascular Outcomes Research central audit database, and the IQIC in low- and middle-income countries, to name a few. These registries have informed congenital interventional practice and have played a part in defining guidelines for intervention. The reliance on registries is becoming more common in pediatric and congenital cardiology, and there is an understanding that the use of such registries must become the standard for evaluating new devices and percutaneous approaches.

## Conclusion

Despite the rapid growth of pediatric interventional cardiology, there are areas that still require attention. In particular, devices specifically designed for pediatric and congenital patients are much needed and have the potential to minimize morbidity and improve outcomes. Several areas of practice in our field lack scientific guidance and could serve as future research directives. Such areas include the standardization of lesion-specific treatment protocols, anti-thrombotic regimens after cardiac catheterization, antibiotic use after device deployment, and long-term outcomes of self-expanding valves in the pulmonary position, to name a few.

## Data Availability

No datasets were generated or analysed during the current study.
